# Below normal birth weight in the Northwest part of Ethiopia

**DOI:** 10.1186/s13104-018-3723-9

**Published:** 2018-08-25

**Authors:** Wale Kumlachew, Nega Tezera, Aklilu Endalamaw

**Affiliations:** 1 Department of Pediatric and Child Health Nursing, College of Health Sciences, Assosa University, Asosa, Ethiopia; 2 Department of Pediatric and Child Health Nursing, School of Nursing, College of Medicine and Health Sciences, University of Gondar, Gondar, Ethiopia

**Keywords:** Birth weight, Newborn, Ethiopia

## Abstract

**Objectives:**

Low birth weight is one of the global agendas that have an impact on the short and long-term health status. A cross-sectional study from March 1 to April 1, 2018 was conducted. 381 mother–newborn pairs were participated. This study aimed to assess the prevalence and associated factors of low birth weight in the Northwest part of Ethiopia.

**Results:**

The prevalence of low birth weight was 14.9% (95% CI 11.7–18.9). Being preterm [adjusted odds ratio (AOR) = 4.1; 95% CI 1.7–9.9], absence of ante-natal care follow-up (AOR = 3.4; 95% CI 1.2–9.5), malaria attack during pregnancy (AOR = 4.2; 95% CI 1.6–11.1), anemia during pregnancy (AOR = 2.6; 95% CI 1.03–7.0), and lack of iron supplementation (AOR = 4.0; 95% CI 1.3–12.6) were predisposing factors to low birth weight. On the other hand, infants born from employed mothers (AOR = 0.1; 95% CI 0.01–0.92) were less likely to born with below normal birth weight. The prevalence of low birth was high as compared to WHO estimation.

## Introduction

Low birth weight (LBW) is one of the reliable indicators and monitoring parameters of maternal and child health programs [1]. According to the world health organization (WHO) definition, LBW considered if weight at birth less than 2500 g [2].

Being below normal birth weight is the greater risk for different severe and life-threatening health complications. Notably, hypothermia, hypoglycemia, birth asphyxia, anemia, impaired nutrition, and respiratory problems are the major complications of LBW [3].

It is possible to prevent LBW prior to its occurrence. Accordingly, WHO sets a 30% reduction of LBW by 2025 through providing affordable, accessible, and appropriate healthcare services [4]. Accessing maternal education, expansion of antenatal care service, promoting planned pregnancy, preventing teenage pregnancy, increasing skilled birth attendants, and improving prenatal care services are implementing to prevent LBW [5].

Despite different healthcare services, LBW is continuing to be one of the important public health problems worldwide. Correspondingly, 15% to 20% of all births worldwide were LBW in 2014 [4]. It is also reported in Nepal (22.3%) [6], Nigeria (6.3%) [7], and Kenya (12.3%) [8]. Similarly, the prevalence of LBW is vary in different geographical areas of Ethiopia; 17.4% in Gondar Ethiopia [9], 11.1% in Southwest Ethiopia [10], and 9.9% in Northern Ethiopia [11]. The social and economic [12], maternal and infant-related factors of LBW are identified from other previous studies [12, 13].

There was no study found in the current study area related to LBW. Even if there are studies in other parts of the country, it cannot be represent the current study area due to the difference in demographic, socio-cultural, and other health coverage status.

Therefore, we aimed to assess the prevalence of low birth weight and its associated factors in the Northwest part of Ethiopia.

## Main text

### Methods

#### Study design, period, setting, and population

An institution based cross-sectional study was employed from March 1 to April 1, 2018, at two hospitals in the delivery clinics of Northwest part of Ethiopia. These hospitals are Assosa and Pawi general hospitals, where the majority of the population of the region gets healthcare services. Based on the current Ethiopian government classification, these hospitals are found in the Benishangul-Gumuz Region, which is located 632 km far from Addis Ababa. Based on the 2007 Census conducted by the Central Statistical Agency of Ethiopia [14], the Benishangul-Gumuz Region has a total population of 784,345. Of which, 398,655 were men and 385,690 were women. A lower percentage (13.51%) of the population were urban inhabitants [15]. According to 2016 EDHS report, the fertility rate of the region was 4.4% [16].

All mother–neonate pairs were the study population.

#### Sample size and sampling technique

The sample size is calculated using single population formula

$$\left( {n\, = \,\left( {za/ 2} \right)^{ 2} {\text{pq}}/{\text{d}}^{ 2} } \right).$$

where n is the desired sample size, Z is the standard normal distribution 1.96, P is the prevalence of low birth weight in Gondar Ethiopia (17.4%) [9], q is the proportion of the target population without the problem (1 − p), d is the 4% margin of error, n = (1.96)^2^(0.174) (0.826)/(0.04)^2^ = 346.

Considering 10% non-response rate, the total sample size is 346 + 35 = 381.

Participants were selected using proportional allocation; 241 samples from Assosa, and 140 from Pawi Hospital from the total of 710 mother–infant pairs. A systematic random sampling technique was conducted. Each participant was selected using (k ≈ 2) after providing a number to pregnant mothers attending delivery room from day one to the end of data collection. In situations, where a respondent did not agree or not met the inclusion criteria, the next random position would be considered.

#### Operational definition

Below normal birth weight: neonates whose birth weight less than 2500 g.

#### Data collection tools and procedures

Data was collected using a structured and pre-tested questionnaire, measurement, and chart review. The questionnaire was adapted from the Ethiopian demographic and health survey [16] and other literature. The questionnaire had three parts. The first part contains socio-demographic characteristics, the second part contains infant-related variables, and the third part contains maternal and obstetric-related variables.

Two diploma midwives and nurses who were working outside the study area collected the data. The weight of the newborn was measured after 30 min of delivery using a balanced weight scale. Maternal height was measured against a wall height scale to the nearest centimeter. Maternal weight was measured by beam balance to the nearest kilogram. Participants’ medical charts were reviewed to take some important variables like maternal hemoglobin level.

#### Data processing and analysis

Firstly, data were checked for completeness and inconsistencies. The collected data was entered into Epi-data version 4.2.0.0. and then exported to STATA version 14.0 for analysis. The socio-demographic distributions of the participants were described using descriptive statistics. Binary logistic regression analysis was applied. The multivariable logistic regression analysis was done for variables with a P-value less than 0.25 in the bivariable analysis. Those variables with P-value ≤ 0.05 were claimed as significantly associated factors of LBW.

#### Ethical considerations

Ethical clearance was obtained from the School of Nursing on behalf of the University of Gondar Institutional Ethical Review Committee. Written permission was taken to both Assosa and Pawi General Hospitals’ manager. Then, each respective manager wrote permission letter to the focal persons. Name or identification number of study participants was not recorded. Participant data was used only for the study purpose.

### Result

#### Maternal socio-demographic characteristics

Three hundred seventy-five mother–neonate pairs have participated with a response rate of 98.4%. Mean age of mothers in this study was 27.19 ± 5.13 years. Of these, 72.53% mothers were between the ages of 20–35 years. About 45.6% of mothers were housewives. Regarding the residence, 33.33% were rural dwellers. The majority (41.87%) of mothers were diploma and above. The majority (30.13%) were Amhara ethnic group (Table 1).

**Table 1:** Maternal demographic characteristics of mothers Assosa and Pawi hospitals, Northwest Ethiopia, 2018

Variable	Frequency	Percent
Maternal age (years)		
15–20	40	10.67
20–35	272	72.53
> 35	63	16.80
Marital status		
Married	265	70.67
Single	54	14.40
Divorced	25	6.67
Widowed	31	8.23
Residence		
Rural	125	33.33
Urban	250	66.67
Educational level		
Uneducated	106	28.27
Up to secondary	112	29.87
Diploma and above	157	41.87
Occupation		
Housewife	171	45.60
Merchant	55	14.67
Employ	109	29.07
Student	40	10.67
Ethnicity		
Amhara	113	30.13
Oromo	69	18.40
Agew	30	8.00
Shinasha	58	15.47
Gumuz	26	6.93
Berta	36	9.60
Mao	19	5.07
Komo	11	2.93
Others	13	3.47

#### Association between LBW and socio-demographic characteristics

From the study participants, 56 were LBW, which makes the prevalence to be 14.93%. Mothers who were employed were significantly associated with LBW (AOR = 0.11; 95% CI 0.01–0.92) (Table 2).

**Table 2:** The association between LBW and maternal demographic characteristics in Assosa and Pawi hospitals, Northwest Ethiopia, 2018

Variable	LBW		NBW		Crude OR (95% CI)	AOR (95% CI)
	No.	%	No.	%		
Maternal age (years)						
15–20	8	14.29	32	10.03	1.59 (0.68, 3.71)	
20–35	37	66.07	235	73.67	1	
> 35	11	19.64	52	16.30	1.34 (0.64, 2.81)	
Marital status						
Married	36	64.29	229	71.79	1	
Single	10	17.86	44	13.79	1.45 (0.67, 3.13)	
Divorced	5	8.93	20	6.27	1.59 (0.56, 4.50)	
Widowed	5	8.93	26	8.15	1.22 (0.44, 3.39)	
Residence						
Rural	20	35.71	105	32.92	1.13 (0.63, 2.05)	
Urban	36	64.29	214	67.08		
Educational level						
Uneducated	20	35.71	86	26.96	3.09 (1.41, 6.75)	1.06 (0.22, 5.06)
Up to secondary	25	44.64	87	27.27	1	1
Diploma and above	11	19.64	146	45.77	3.81 (1.79, 8.13)	1.72 (0.46, 6.43)
Occupation						
Housewife	31	55.36	140	43.89	1	1
Merchant	8	14.28	47	14.73	0.77 (0.33, 1.79)	0.77 (0.23, 2.61)
Employ	2	3.57	107	33.54	0.08 (0.02, 0.36)	0.11 (0.01, 0.92)
Student	15	26.79	25	7.84	2.71 (1.28, 5.73)	1.03 (0.24, 4.38
Ethnicity						
Amhara	16	28.57	97	30.41	1	
Oromo	13	23.21	56	17.55	1.41 (0.63, 3.14)	
Agew	3	5.36	27	8.46	0.67 (0.18, 2.48)	
Shinasha	9	16.07	49	15.36	1.11 (0.46, 2.70)	
Gumuz	5	8.93	21	6.58	1.44 (0.48, 4.38)	
Berta	6	10.71	30	9.40	1.21 (0.44, 3.38)	
Mao	2	3.57	17	5.33	0.71 (0.15, 3.39)	
Komo	1	1.79	10	3.13	0.61 (0.07, 5.06)	
Others	1	1.79	12	3.76	0.51 (0.06, 4.16)	

#### Association of obstetric history of mothers and newborn-related factors with LBW

Mother who did not have ANC follow-up (AOR = 3.45; 95% CI 1.25–9.55), mother who did not get iron tablet during pregnancy (AOR = 4.06; 95% CI 1.31–12.61), malaria attack during pregnancy (AOR = 4.28; 95% CI 1.65–11.14), and anemia during pregnancy (AOR = 2.69; 95% CI 1.03–7.01) are identified associated factors with LBW. Regarding neonatal-related factors, being preterm (AOR = 4.15, 95% CI 1.74–9.89) was associated with LBW (Table 3).

**Table 3:** The association between LBW and maternal obstetric and neonate-related factors in Assosa and Pawi hospitals, Northwest Ethiopia, 2018

Variable	LBW		NBW		Crude OR (95% CI)	AOR (95% CI)
	No.	%	No.	%		
Parity						
Multi-para	41	73.21	239	74.92	1	
Primi-para	15	26.79	80	25.08	1.09 (0.57, 0.08)	
Maternal MUAC						
< 23 cm	9	16.07	41	12.85	1.30 (0.59, 2.85)	
≥ 23 cm	47	83.93	278	87.15	1	
Pregnancy						
Singleton	48	85.71	270	84.64	1	
Multiple	8	14.29	49	15.36	0.92 (0.41, 2.06)	
History of abortion						
Yes	6	10.71	25	7.84	1.41 (0.55, 3.61)	
No	50	89.29	294	92.16	1	
Number of ANC						
None	33	58.93	42	13.17	9.46 (5.07, 17.65)	3.45 (1.25, 9.55)
One and above	23	41.07	277	86.83	1	1
Iron tablet						
Not taken	27	48.21	25	7.84	14.64 (7.09, 30.22)	4.06 (1.31, 12.61)
Up to 1 month	11	19.64	50	15.64	2.98 (1.33, 6.70)	2.43 (0.83, 7.17)
More than 2 months	18	32.14	244	76.49	1	1
Malaria						
Yes	35	62.50	33	10.34	14.44 (7.54, 27.67)	4.28 (1.65, 11.14)
No	21	37.50	286	89.66	1	1
PIH						
Yes	3	5.36	14	4.39	1.23 (0.34, 4.4)	
No	53	94.64	305	95.61	1	
Anemia						
Yes	28	50.00	37	11.60	7.62 (4.08, 14.25)	2.69 (1.03, 7.01)
No	28	50.00	282	88.40	1	1
Previous LBW						
Yes	7	12.50	29	9.09	1.43 (0.59, 3.44)	
No	49	87.50	290	90.91	1	
Confirmed DM						
Yes	1	1.79	6	1.88	0.95 (0.11, 8.03)	
No	55	98.21	313	98.12	1	
Maternal weight						
< 50 kg	18	32.73	32	10.09	4.33 (2.21, 8.48)	2.37 (0.89, 6.35)
≥ 50 kg	38	67.27	287	89.91	1	1
Maternal height						
< 1.50 m	8	14.29	34	10.66	1.40 (0.61, 3.20)	
≥ 1.50 m	48	85.71	285	89.34	1	
Substance use						
Yes	0	0.00	0	0.00		
No	56	14.93	319	85.07		
Maternal abuse						
Yes	0	0.00	0	0.00		
No	56	14.93	319	85.07		
Gestational age (weeks)						
< 37	32	57.14	57	17.87	6.13 (3.36, 11.19)	4.15 (1.74, 9.89)
≥ 37	24	42.86	262	82.13	1	1
Sex of neonate						
Male	20	35.71	172	53.92	1	1
Female	36	64.29	147	46.08	2.11 (1.17, 3.80)	1.76 (0.75, 4.12)

## Discussion

Low birth weight prevalence varies in different geographical areas. Estimating the burden of LBW in the current study area was found to be relevant for the regional policy implementation and healthcare resources allocation. Accordingly, the prevalence of LBW was14.9% (95% CI 11.7–18.9). The finding of the current study was comparable with a study conducted in Tigray Ethiopia (14.6%) [17], Gondar Ethiopia (17.4%) [9], and southern Ethiopia (17.88%) [18].

On the other hand, it was higher than other Ethiopian settings, like Axum (9.9%) [11] and Jimma Ethiopia (11.02%) [10]. This might be due to the current study setting was a hospital setting where many chronic cases were referred from other health institutions but the others were community-based studies. Besides, the variation might be due to in the current study area was not well accessible for comprehensive health programs [16] and further due to cultural issues, in which one of the ethnic groups “Gumuz” population health seeking behaviors are poor. Besides, in “Gumuz” ethnic group, pregnant woman give birth alone and/or get away from around the peoples. The high malaria burden in the study area might be attributed to a high prevalence of LBW.

The current finding also higher than the finding from other African settings, like Nigeria [7], the United States of America [19], and Canada [20]. The possible explanation might be due to the difference in the level of antenatal care follow-up, the burden of food insecurity, the educational status of the community, cultural malpractices, and the high burden of co-morbid illness in Ethiopia.

Below normal birth weight is associated with the infant, maternal and obstetric related or environment and behavioral factors. Gestational age, malaria attack during pregnancy, anemia during pregnancy, the absence of ANC follow-up, and absence of iron intake during pregnancy were associated with LBW.

Regarding gestational age, preterm neonates were 4.2 times more likely to be LBW than term neonates. This finding was in agreement with a study done in Bale Ethiopia [21]. This might be due to the fact that as the gestational age of the fetus is lowered, prematurity and inadequate production of subcutaneous fat is prevalent [22].

The absence of ANC follow-up during pregnancy was another variable. Women had not ANC follow-up were 3.5 times more likely to give LBW baby than women who had ANC follow-up. The possible reason might be due to, in those who had no ANC follow-up, unable to detect early major health problems, pregnancy danger sign, and cultural malpractices that can affect the birth outcome of the neonate [23]. The other possible explanation might be those mothers who had no ANC visit during pregnancy may not receive iron supplementation and counseling about lifestyle and nutrition like food diversification during pregnancy to nourish the fetus for better growth and development during fetal growth [23].

Those mothers had history of malaria attack during pregnancy were 4.3 times more likely to deliver LBW baby than mothers had no history of malaria attack during pregnancy. This result is consistent with a study done in Gondar Ethiopia [9] and sub-Saharan Africa [24]. This might be due to long-standing infections during pregnancy has a direct effect to limit fetal growth. One of the pathological effects of malaria is prematurity. This is due to placental infection with malaria causes the placenta to carry antibodies, cytokines, and macrophages which later causes early initiation of labor [25].

This study found anemia was one of the risk factors of LBW. Anemic mothers had 2.7 times more likely of getting a LBW baby than non-anemic mothers. This finding is in agreement with a study done in Sudan [26]. This is because the lack of hemoglobin due to anemia leads to impaired nutrient and oxygen transport to the fetus through the maternal placenta and the normal fetal growth in the utero may compromise as a result. Moreover, anemia is one of the chronic outcomes of systemic infections, which had a direct effect on the intrauterine growth retardation [27].

Mothers who did not take iron during pregnancy were 4.1 times more likely of getting LBW baby than who took iron. A study in India [28] was agreed with this finding. Iron and folic acid can prevent the occurrence of anemia and has a positive effect on the supplementation of oxygen and nutrient to the fetus. The absence of utilizing iron and folic acid contribute to poor organ development [29]. Besides, according to the current study, mothers being employed either government or other private sectors were 89% protective to LBW.

This study found that the prevalence of LBW was still high compared with WHO goal by 2025 which is a 3% reduction every year from 2012 to 2025. Being preterm, lack of ANC follow-up, malaria attack during pregnancy, lack of iron supplementation, and employed mothers were associated factors of LBW.

## Limitation

This study shares the limitation of cross-sectional study design and therefore, it does not show the seasonal variation of LBW.

## Abbreviations

ANC: ante-natal care
AOR: adjusted odds ratio
EDHS: Ethiopian Demographic and Health Survey
LBW: low birth weight
MUAC: Mid Upper Arm Circumference
PIH: pregnancy induced hypertension
WHO: World Health Organization
